# Supplementary material for: Content-rich biological network constructed by mining PubMed abstracts
Source: BMC Bioinformatics. 2004 Oct 8;5:147. doi: 10.1186/1471-2105-5-147 (PMC528731; doi:10.1186/1471-2105-5-147)
Supplement: Additional File 2 — The original results of the above study (non-essential files are deleted to keep the file size under the limit set by BMC bioinformatics). [file 1471-2105-5-147-S2.bz2 › chilibotAdditionalFile2/dip20/116ID8645696E460/html/ATPH_ATPC.html]

 


 **ATPH** and **ATPC** 
  
Found 201 abstracts in PubMed, retrieved 20.  
 

 What does Google say? 
 PDF only 
| .edu only 

---

**Interactive relationship** (e.g. stimulation, inhibition, etc)

**Neutral relationship**- Adipocyte lipid binding protein  [ **ATPC** ]   [ **ATPH** ]  ALBP or aP2 is an intracellular fatty acid binding protein which is found in adipocytes and macrophages and binds a large variety of intracellular lipids with high affinity.  Ref: 12794068 J Biol Chem< MedlineTA>J Biol Chem,
- Brain lipid binding protein  [ **ATPC** ]   [ **ATPH** ]  in axon Schwann cell interactions and peripheral nerve tumorigenesis.  Ref: 12612091 Mol Cell Biol, 2003

**Non-interactive relationship** (e.g. studied together, co-existance, homology, etc.)

- Apolipoprotein E apoE is a lipid binding protein  [ **ATPC** ]   [ **ATPH** ]  that plays an important role in tissue repair following brain injury.  Ref: 12793762 J Radiat Res Tokyo , 2002
- The zebrafish CRABP exhibits highest sequence identity to the pufferfish CRABPIIa 83% and CRABPIIb 79%, and human CRABPII 74% than to any other member of the intracellular lipid binding protein  [ **ATPC** ]   [ **ATPH** ]  ILBP family.  Ref: 12853146 Gene, 2003
- Role of adipocyte lipid binding protein  [ **ATPC** ]   [ **ATPH** ]  ALBP and acyl coA binding protein ACBP in PPAR mediated transactivation.  Ref: 12479581 Mol Cell Biochem, 2002
- Results Resection had no effect on the mRNA expression for the early response genes, for proglucagon, or for the ileal lipid binding protein  [ **ATPC** ]   [ **ATPH** ]  ILBP, but was associated with reduced jejunal ornithine decarboxylase ODC mRNA and with reduced jejunal mRNA for the liver fatty acid binding protein L FABP.  Ref: 12428071 Digestion, 2002
- Members of the phylogenetically related intracellular lipid binding protein  [ **ATPC** ]   [ **ATPH** ]  iLBP are characterized by a highly conserved tertiary structure, but reveal distinct binding preferences with regard to ligand structure and conformation, when binding is assessed by the Lipidex method removal of unbound ligand by hydrophobic polymer or by isothermal titration calorimetry, a true equilibrium method.  Ref: 12479567 Mol Cell Biochem, 2002
- From this analysis, we have identified adipocyte lipid binding protein  [ **ATPC** ]   [ **ATPH** ]  ALBP aP2 as a gene that is highly upregulated in foam cells in response to oxLDL.  Ref: 12417276 Atherosclerosis, 2002
- Treating differentiating 3T3 L1 preadipocytes with t10c12 CLA and conjugated nonadecadienoic acid CNA, a 19 carbon CLA cognate resulted in decreased intracellular triglyceride accumulation and mRNA levels of the adipogenic gene fatty acid synthase and adipocyte lipid binding protein  [ **ATPC** ]   [ **ATPH** ] .  Ref: 12670481 Biochem Biophys Res Commun, 2003
- The binding specificity of the protein is not known based on the high sequence and structural similarity with an orthologous protein, ileal lipid binding protein  [ **ATPC** ]   [ **ATPH** ] , it is suggested that bile acids may be the putative ligands.  Ref: 12652125 J Biomol NMR, 2003
- Using cDNA microarrays, we found that brain lipid binding protein  [ **ATPC** ]   [ **ATPH** ]  BLBP is elevated in an EGFR positive subpopulation of Nf1 mutant mouse Schwann cells Nf1 TXF that grows away from axons.  Ref: 12612091 Mol Cell Biol, 2003
- This is a 136 residue, 10 stranded, antiparallel beta barrel protein with seven beta hairpins and is a member of the intracellular lipid binding protein  [ **ATPC** ]   [ **ATPH** ]  iLBP family.  Ref: 12834350 Biochemistry, 2003
- The relative ligand binding preference of the murine ileal lipid binding protein  [ **ATPC** ]   [ **ATPH** ] .  Ref: 12651103 Protein Expr Purif, 2003
- Salt modulates the stability and lipid binding affinity of the adipocyte lipid binding protein  [ **ATPC** ]   [ **ATPH** ] .  Ref: 12794068 J Biol Chem< MedlineTA>J Biol Chem, 2003
- In parallel, a rapid and dramatic decrease in the level of mRNA encoding for several adipocyte specific proteins such as adipocyte lipid binding protein  [ **ATPC** ]   [ **ATPH** ] , hormone sensitive lipase, lipoprotein lipase, and peroxisome proliferation activating receptor gamma2 was observed in isolated adipocytes.  Ref: 12734776 Horm Metab Res, 2003
- Among fatty acids, mILBP preferred species that had longer chain length and increased saturation, similar to other members of the intracellular lipid binding protein  [ **ATPC** ]   [ **ATPH** ]  family.  Ref: 12651103 Protein Expr Purif, 2003
- Two fatty acid binding proteins FABPs are expressed in adipose tissue, adipocyte lipid binding protein  [ **ATPC** ]   [ **ATPH** ]  ALBP and keratinocyte lipid binding protein  [ **ATPC** ]   [ **ATPH** ]  KLBP.  Ref: 12479574 Mol Cell Biochem, 2002
- Insights into the bile acid transportation system the human ileal lipid binding protein  [ **ATPC** ]   [ **ATPH** ]  cholyltaurine complex and its comparison with homologous structures.  Ref: 12486725 Proteins, 2003
- The small intestine contains three distinct proteins belonging to the intracellular lipid binding protein  [ **ATPC** ]   [ **ATPH** ]  family the liver type fatty acid binding protein L FABP, the intestinal fatty acid binding protein I FABP and the ileal lipid binding protein  [ **ATPC** ]   [ **ATPH** ]  ilbp.  Ref: 12479571 Mol Cell Biochem, 2002
- The ileal lipid binding protein  [ **ATPC** ]   [ **ATPH** ]  ILBP, a member of the intracellular lipid binding protein  [ **ATPC** ]   [ **ATPH** ]  family, is a 14 kDa protein that has bile and fatty acids as possible physiological ligands.  Ref: 12651103 Protein Expr Purif, 2003
- We have also shown that the adipocyte lipid binding protein  [ **ATPC** ]   [ **ATPH** ]  ALBP, the keratinocyte lipid binding protein  [ **ATPC** ]   [ **ATPH** ]  KLBP and the acyl CoA binding protein ACBP exhibit a prominent nuclear localization in differentiating 3T3 L1 adipocytes.  Ref: 12479581 Mol Cell Biochem, 2002
- For example, it has been reported that some beta proteins in the intracellular lipid binding protein  [ **ATPC** ]   [ **ATPH** ]  family fold through quite different pathways Burns et al.  Ref: 12784211 Proteins, 2003
- Each protein produced a characteristic shift in peak fluorescence emission of DAUDA, and one Ce FAR 5 produced a shift greater than has been observed previously for any lipid binding protein  [ **ATPC** ]   [ **ATPH** ] .  Ref: 12502713 J Biol Chem, 2003
- First, Notch1 activation by neuronal contact induces the glial expression of the brain lipid binding protein  [ **ATPC** ]   [ **ATPH** ]  BLBP and erbB2 genes.  Ref: 12853432 J Neurosci, 2003
- The adipocyte lipid binding protein  [ **ATPC** ]   [ **ATPH** ]  ALBP aP2 gene facilitates foam cell formation in human THP 1 macrophages.  Ref: 12417276 Atherosclerosis, 2002
- Here we report the solution structure of the human ileal lipid binding protein  [ **ATPC** ]   [ **ATPH** ]  ILBP in free form and in complex with cholyltaurine.  Ref: 12486725 Proteins, 2003
- PPARgamma protein level was decreased in a time and concentration dependent manner, and DHEA significantly reduced mRNA levels of PPARgamma, adipocyte lipid binding protein  [ **ATPC** ]   [ **ATPH** ] , and sterol regulatory element binding protein, but not CCAAT enhancer binding protein alpha.  Ref: 12488352 Endocrinology, 2003
